# The development of paranasal sinuses in patients with cystic fibrosis: sinuses volume analysis

**DOI:** 10.1007/s00405-023-08236-x

**Published:** 2023-10-26

**Authors:** Agata Kaluzna-Mlynarczyk, Beata Pucher, Jakub Sroczynski, Michal Kotowski, Katarzyna Jonczyk-Potoczna, Jarosław Szydlowski

**Affiliations:** 1 Department of Pediatric Otolaryngology, Institute of Pediatrics, Poznan University of Medical Sciences, Szpitalna 27/33, 60-572 Poznań, Poland; 2 Doctoral School, Poznan University of Medical Sciences, Bukowska 70, 60-812 Poznań, Poland; 3 Department of Pediatric Radiology, Institute of Pediatrics, Poznan University of Medical Sciences, Szpitalna 27/33, 60-572 Poznań, Poland

**Keywords:** Children, Cystic fibrosis, Paranasal sinuses development, Pediatic otorynolaryngology, Sinuses volume

## Abstract

**Background:**

Cystic fibrosis (CF) is a severe systemic disease that affects many aspects of patients’ lives. It is known that the progression of the disease adversely affects lower and upper airways including the paranasal sinuses. However, its impact on sinus development in the pediatric population is not fully examined. The purpose of this study was to evaluate the development of the paranasal sinuses in a pediatric population with CF and compare it to a control group consisting of healthy children.

**Methods:**

The results of computed tomography (CT) scans of children with the disease and the control group were evaluated. The study included 114 CT images of children in the study group and 126 images of healthy children aged 0–18 years. The volumes of maxillary, frontal, and sphenoid sinuses were analyzed. The obtained results were compared with those of the control group and analyzed statistically.

**Results:**

The volume and the development of the paranasal sinuses in both groups increased with age, but statistically significant differences were found between the study and the control group.

**Conclusions:**

The obtained results provide valuable knowledge regarding the impact of the CF on sinuses development. Also, they may be important in understanding the progression of the disease and its influence on the quality and length of life of patients. The results may contribute to enhanced diagnostics and have implications for improving therapy for patients with chronic sinusitis associated with CF.

**Supplementary Information:**

The online version contains supplementary material available at 10.1007/s00405-023-08236-x.

## Background

Cystic fibrosis (CF) is a severe systemic genetic disease. It is caused by a mutation of the gene encoding the CFTR (cystic fibrosis transmembrane conductance regulator) membrane canal protein, responsible for transporting chloride ions. This results in impaired transporting of chloride ions and water, which subsequently leads to the production of thick secretions in the respiratory tract, gastrointestinal tract, and reproductive system [1, 2]. The secretion–transporting cilia located in each of these systems are unable to manage the high density mucus resulting in reduced mucociliary clearance. This causes symptoms from the respiratory system (recurrent and chronic pneumonia, bronchitis, obstructive bronchitis, recurrent atelectasis, bronchial distension and dilatation, chronic sinusitis, and nasal polyps), digestive system (stinky, fatty stools, digestive disorders caused by thick secretions obstructing the pancreatic ducts, recurrent pancreatitis, cholelithiasis, salivary gland disorders, and meconium obstruction of the intestines in the neonatal period), and the reproductive system (increased density of cervical mucus in women, obstruction, and aplasia of the vas deferens in men) [2–4]. As of now, it is an incurable disease, leading to premature death, which usually occurs in the 3rd decade of life. CF can only be treated symptomatically thus improving the quality and length of life [1–3].

According to a recent report by the European Cystic Fibrosis Society Patient Registry, there are more than 52,000 patients diagnosed with cystic fibrosis in 40 participating European countries [5]. Among them, 46.9% are children under the age of 18. In Poland, a total of 1332 patients were registered alive as of 31 December 2020, 889 (66.74%) of whom are children.

It is widely known that the development of the paranasal sinuses can be impaired in cases of chronic inflammation [6]. However, the influence of the disease on the development of the paranasal sinuses and therefore on their volume remains unclear. Previously published analyses were performed on small groups of patients which often makes it impossible to subdivide, evaluate and conclude across age groups [7]. Such results have a high risk of error and are often statistically insignificant. In our study, we took into account the important aspect of age grouping, making it possible to determine the developmental norms for pediatric patients with CF. We adopted the research hypothesis that the disease negatively affects the development of the paranasal sinuses by slowing their pneumatization and volume expansion with age, which is the norm in healthy children. In our study, we proposed an analysis involving a large number of patients along with age grouping to enable an accurate assessment of sinus development. Comparing the development of the sinuses in patients with CF and healthy children will bring an important contribution to the diagnosis and assessment of the severity of sinus symptoms in affected patients.

## Materials and methods

### Study design

A retrospective evaluation of the CT scans of the paranasal sinuses in patients diagnosed with CF was performed. All patients qualified for the study were treated in the Karol Jonscher Clinical Hospital of the Poznan University of Medical Sciences in the years 2009–2022. Each participant had at least one CT examination of the paranasal sinuses. The study included the assessment of the volumes of maxillary, sphenoid and frontal sinuses. The imaging was performed with the use of a 64-slice Siemens CT scanner (with 128-slice reconstruction capability), model Somatom Definitions AS, according to standard- or low-dose protocol. CT scans performed outside the hospital were also included if provided by the patients or their legal guardians during the mentioned period.

### Study group

In total, 114 test results from 78 patients diagnosed with cystic fibrosis were included in the research group.

The control group consisted of healthy children who underwent a CT examination of the sinuses or the head for reasons other than sinusitis or qualifying for paranasal sinus surgery. The most common indications for imaging in the control group were head and craniofacial injuries (unless sinuses were affected), headaches and dizziness, convulsions, qualification for cochlear implantation, chronic otitis media, lacrimal duct obstruction, facial nerve palsy, nasal dorsum cysts, and choanal atresia.

In total, 126 studies from 126 patients were included in the control group.

Exclusion criteria for the study group and the control group were as follows: age above 18 years old at the moment of the CT examination, inability to evaluate the CT scan due to a recording error, inability to upload scans to the software used for sinus evaluation, incomplete scope of the study (partial imaging of the sinuses in the CT scans), artifacts preventing the measurement of sinus volume (e.g., due to patient movement during imaging), diseases that may affect the development of paranasal sinuses, including children with skull deformities (e.g., craniosynostosis) and congenital malformation syndromes with facial bone deformity (e.g., trisomy of chromosome 21, CHARGE syndrome).

**Fig. 1:**
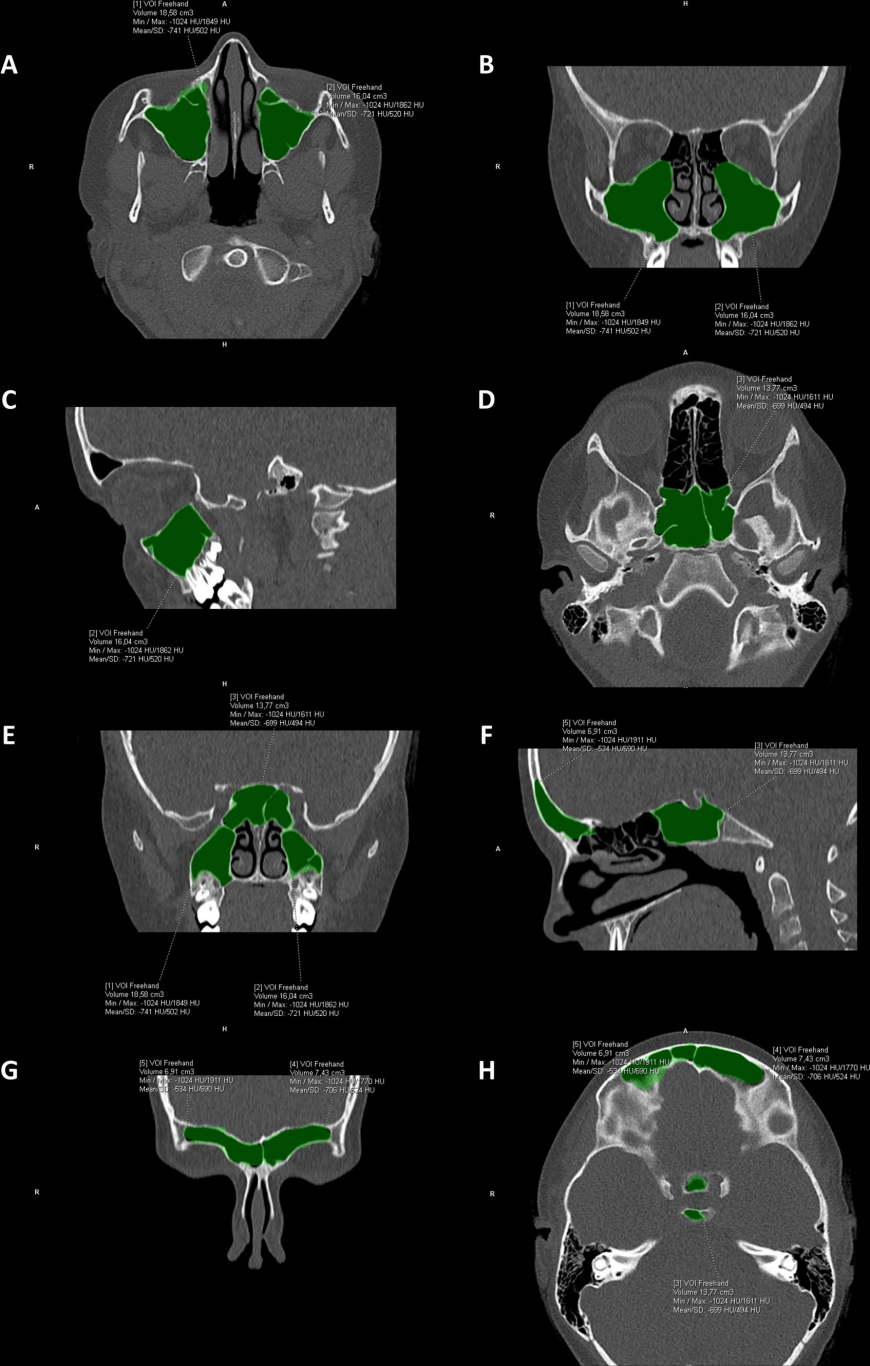
Syngo Via image of paranasal sinuses with marked volume measurements (green), **A** axial plane (maxillary sinuses), **B** coronal plane (maxillary sinuses), **C** sagittal plane (left maxillary sinus), **D** axial plane (sphenoid sinus), **E** coronal plane (sphenoid sinus and maxillary sinuses), **F** sagittal plane (right frontal sinus and sphenoid sinus), **G** coronal plane (frontal sinuses), **H** axial plane (frontal sinuses and sphenoid sinus)

The analysis was performed using the Siemens Syngo Via workstation (Syngo Via Software No. VD12A). The volume of maxillary, sphenoid and frontal sinuses were assessed using standard software for MPR (multiplanar reformated reconstruction) image evaluation. Ethmoid sinuses were excluded from the analysis due to the difficulties in determining their boundries in patients prior to sinus surgery and in patients after ethmoidectomy. The method that was used to evaluate the volume was the VOI Freehand, as this method requires manual delineation of sinus boundaries in computed tomography in one plane on several (at least 10) levels. Based on the contours, the volume of the entire paranasal sinus can be automatically calculated. During the measurements, the outline was always made in cross section. After the final measurement, it was possible to visually assess the correctness of the sinus volume (color coverage)—Fig. 1.

The boundaries of the sphenoid sinuses are sharp and always clearly visible. For the maxillary sinuses, the boundaries are also easy to determine in previously un-operated patients. In patients who have previously undergone antrotomy, the border of the medial wall of the maxillary sinus was established as a visible bone border, and at the level of the antrostomy, a line connecting the anterior and posterior border of the surgical opening. The frontal sinuses pass freely through the mouth of the sinus and the frontal hiatus into the nasal cavity. During the examination, the lower limit of the frontal sinus was assumed at the level of its orifice in the plane of the frontonasal suture.

The sinuses on the right and left sides were not analyzed separately. Symmetrical sinuses (maxillary and frontal) were analyzed in a common group without distinguishing sides (CF = 228 cases, control = 252 cases). The sphenoid sinus as an individual was analyzed in a smaller data group (CF = 114 cases, control = 126 cases).

The obtained results were divided into 6 age groups: I—under 3 years old, II—from 3 to 5 years old (≥ 3 and < 6 years old), III—from 6 to 8 years old (≥ 6 and < 6 years old), IV—from 9 to 11 years old (≥ 9 and < 12 years old), V—from 12 to 14 years old (≥ 12 and < 15 years old), VI—from 15 to 17 years old (≥ 15 and < 18 years old). Then, a comparison between each of the research groups and the corresponding control group was performed.

The results were subjected to statistical analysis with the use of TIBCO Statistica 13 and PQStat Software PQStat v. 1.8.4. The Student’s *t*-test or Cochran–Cox test was used for a normal distribution depending on the variance. In the absence of a normal distribution, calculations were performed using Mann–Whitney test. The *α *value used was 0.05.

## Results

The study and the control group sizes for each sinus and age group are summarized and shown in Table 1.

**Table 1:** Summary of study and control group sizes for each sinus and age group including mean, median, and *p* value

Group	*N*	Mean [cm^3^]	Median [cm^3^]	*p* value
Maxillary sinus				
< 3-year-old CF	6	4.86	2.25	
< 3-year-old control	42	2.27	2.39	
3–5-year-old CF	44	8.70	8.82	**0.0003**
3–5-year-old control	42	6.90	6.79	
6–8-year-old CF	40	8.11	7.95	**0.0000**
6–8-year-old control	42	10.95	10.86	
9–11-year-old CF	38	10.26	10.49	**0.0000**
9–11-year-old control	42	15.33	15.14	
12–14-year-old CF	58	10.13	9.93	**0.0000**
12–14-year-old control	44	18.72	18.82	
15–17-year-old CF	42	11.32	11.06	**0.0000**
15–17-year-old control	40	22.10	21.61	
Sphenoid sinus				
< 3-year-old CF	3	0.19	0.00	
< 3-year-old control	21	0.30	0.33	
3–5-year-old CF	22	3.24	2.40	0.8937
3–5-year-old control	21	3.24	2.85	
6–8-year-old CF	20	2.38	2.48	**0.0000**
6–8-year-old control	21	7.17	6.88	
9–11-year-old CF	19	2.83	1.65	**0.0000**
9–11-year-old control	21	12.60	11.75	
12–14-year-old CF	29	2.91	2.33	**0.0000**
12–14-year-old control	22	13.68	14.10	
15–17-year-old CF	21	3.16	2.12	**0.0000**
15–17-year-old control	20	15.59	14.95	
Frontal sinus				
< 3-year-old CF	6	0.10	0.00	
< 3-year-old control	42	0.03	0.00	
3–5-year-old CF	44	0.38	0.21	0.4362
3–5-year-old control	42	0.33	0.12	
6–8-year-old CF	40	0.44	0.23	**0.0000**
6–8-year-old control	42	1.56	1.41	
9–11-year-old CF	38	1.49	1.13	**0.0000**
9–11-year-old control	42	4.08	3.94	
12–14-year-old CF	58	2.07	1.54	**0.0000**
12–14-year-old control	44	5.22	4.47	
15–17-year-old CF	42	2.98	2.35	**0.0000**
15–17-year-old control	40	6.60	5.15	

*N* number of cases; The statistically significant p-values are bolded

**Fig. 2:**
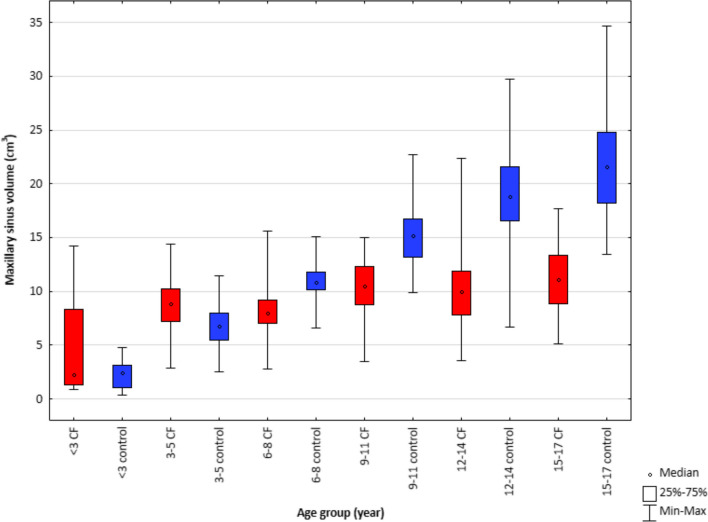
Maxillary sinus volume in children with cystic fibrosis and healthy children in 6 age groups

**Fig. 3:**
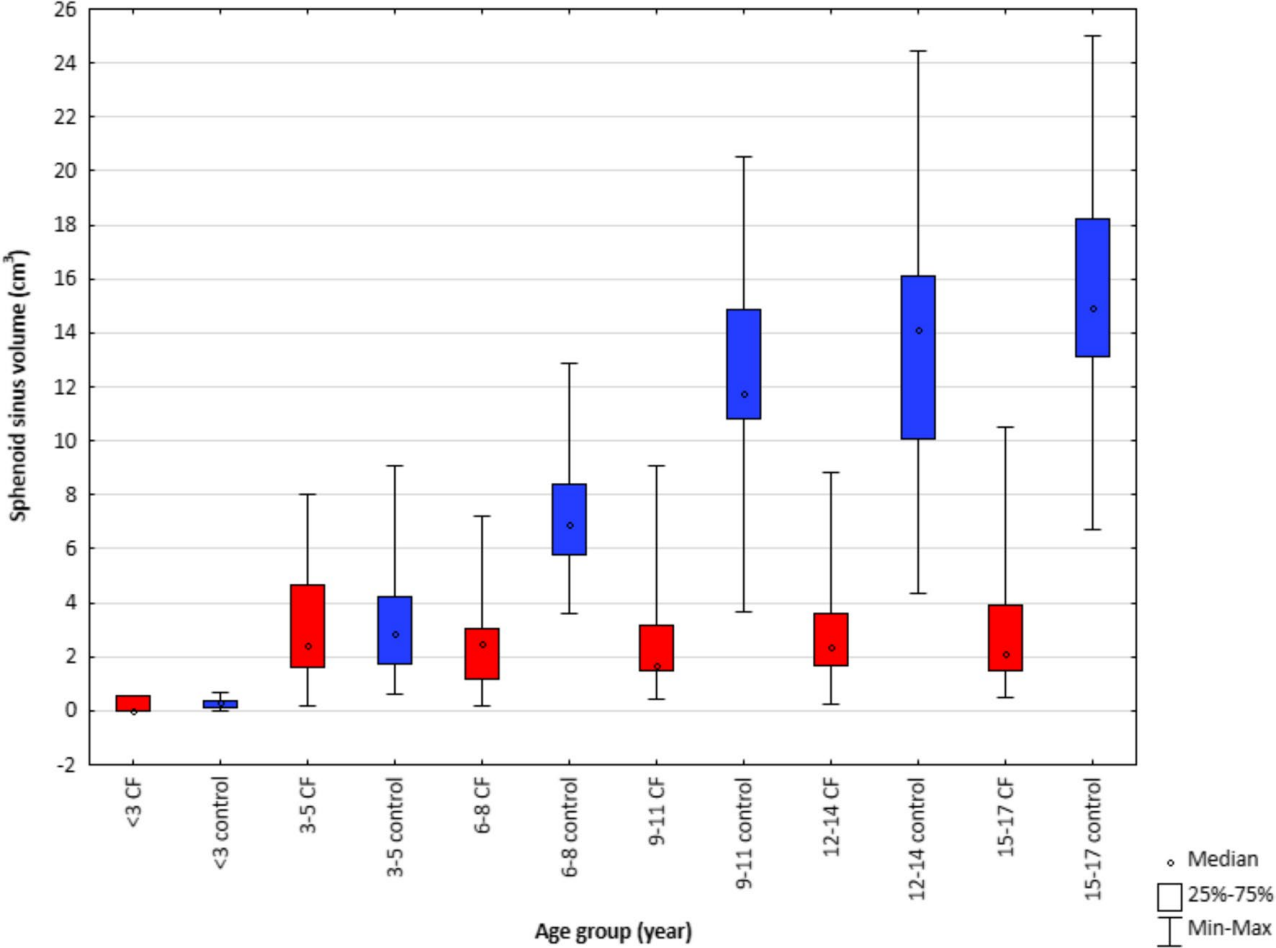
Sphenoid sinus volume in children with cystic fibrosis and healthy children in 6 age groups

**Fig. 4:**
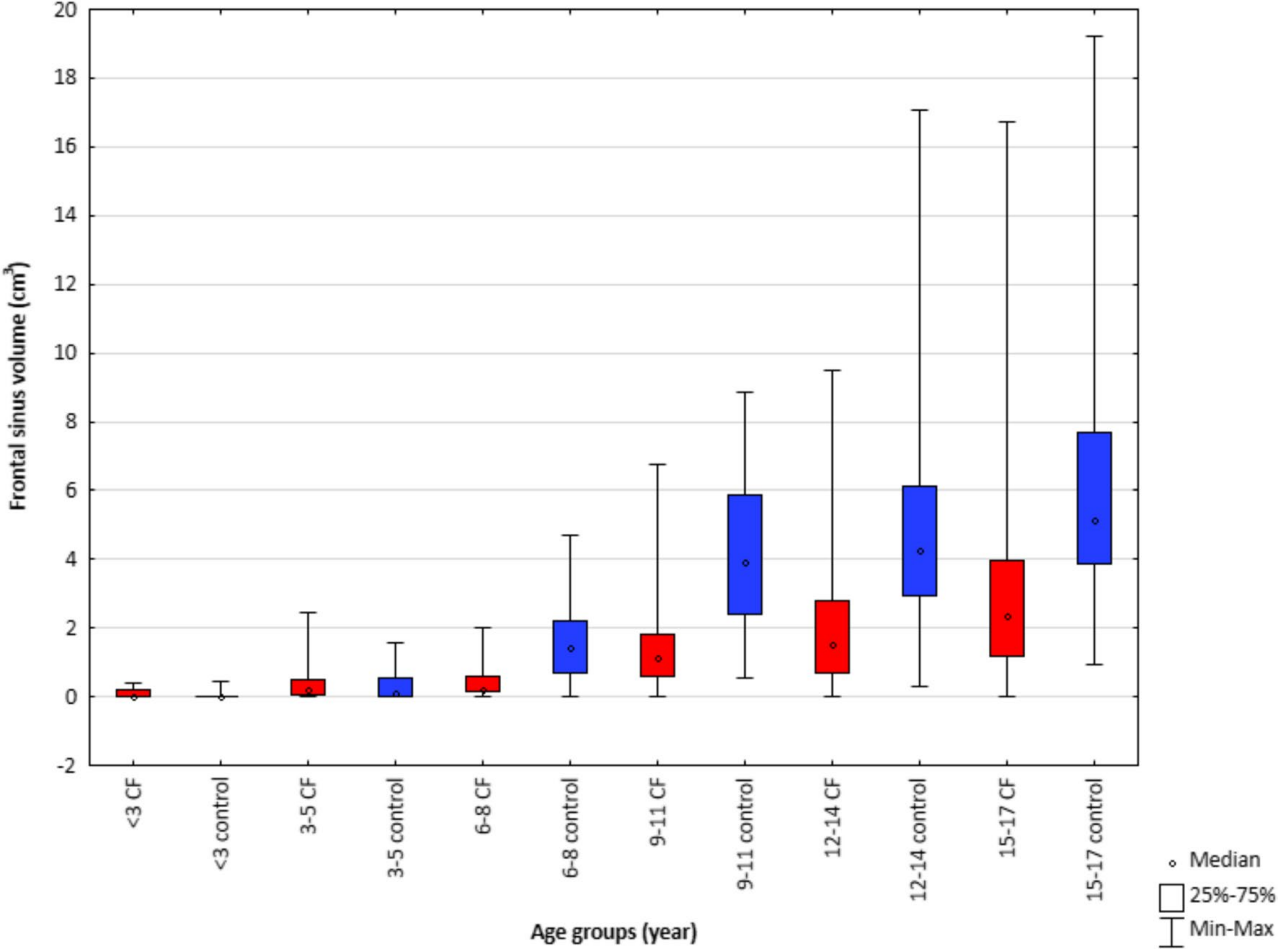
Frontal sinus volume in children with cystic fibrosis and healthy children in 6 age groups

**Fig. 5:**
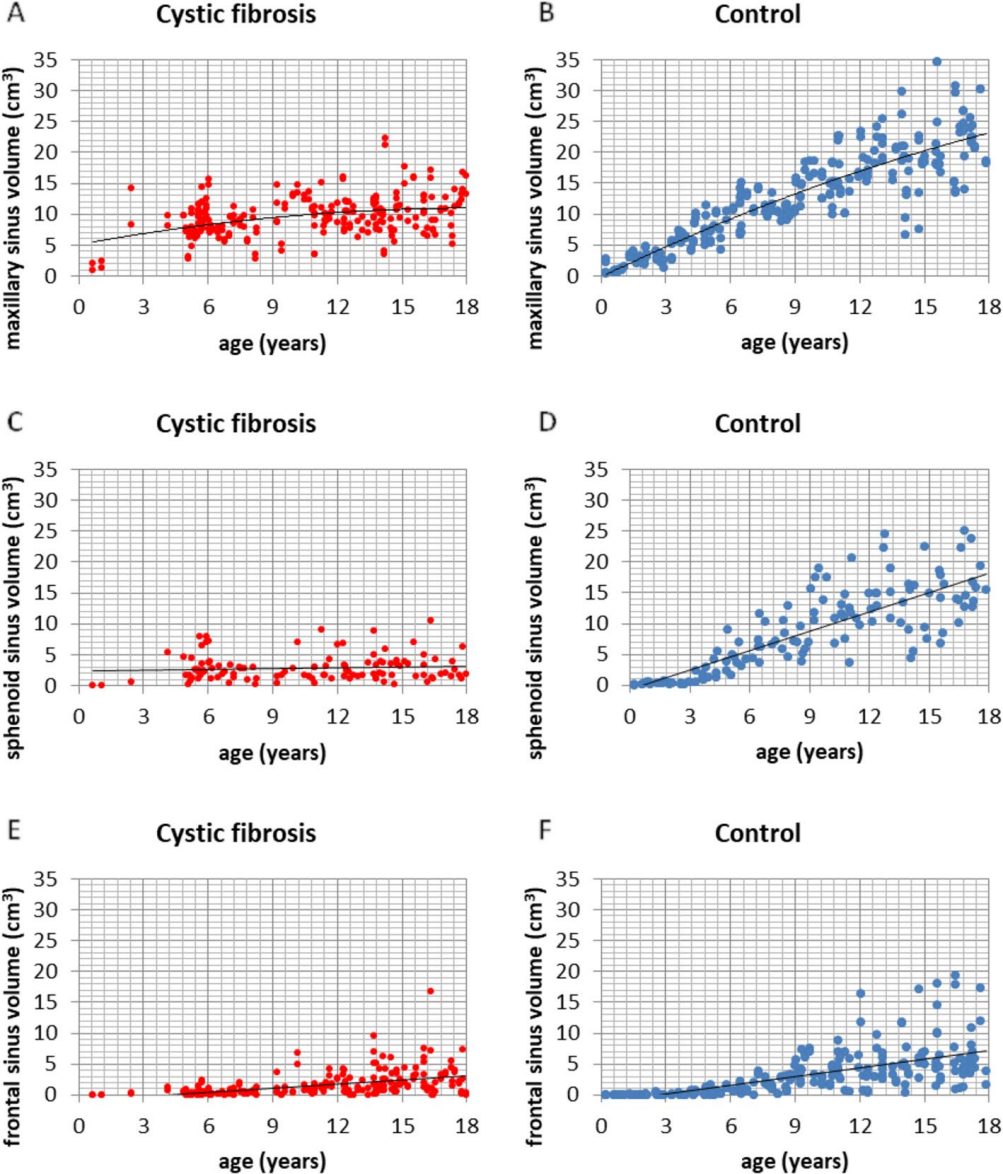
The distribution of sinus volume scores according to age (Spearman Rank Correlation): **A** maxillary sinuses in the CF group, **B** maxillary sinuses in the control group, **C** sphenoid sinuses in the group with CF, **D** sphenoid sinuses in the control group, **E** frontal sinuses in the group with CF, **F** frontal sinuses in the control group

For the CF group aged up to 3 years, it was not possible to perform statistical analysis due to the small number of patients in the group. Nevertheless, the results obtained for this group were included in the graphs as well (Figs. 2, 3, 4 and 5a, c, e).

Some interesting differences were found between the volume of the maxillary sinuses when comparing the study and control groups. These differences are statistically significant for age groups II, III, IV, V, and VI (*p = *0.000). For group II, there is a statistically larger sinus volume in the CF group compared to the control group. For the older age groups, the opposite trend can be seen—the sinus volume in the study group is smaller compared to the control (Fig. 2). The trend line is increasing with age for both the study and control groups, but the trend is significantly more marked for the group of healthy children (Fig. 5a, b).

Based on the results, an increase in maxillary sinus volume with age can be found in a group of healthy children. The increase is also observed for the group of children with CF. However, comparing the two groups with each other, it is noticable that the growth of the maxillary sinuses in children with impaired mucociliary drainage occurs at a lower rate. The larger volume of the maxillary sinus in patients with CF for the age group of 3–6 years is most likely due to the presence of lesions (dilatation of the sinus due to mucosal hypertrophy, the presence of polyps inside the sinus and impaired drainage of secretion). In older children, underdevelopment of the sinus dominates over pathological changes, which does not affect further enlargement of the volume.

The volume of the sphenoid sinus is significantly higher in the control group than in the CF group for children of 6 years of age and older (*p = *0.000) (Fig. 3). The volume increase with age is also noticeable for the CF group but is lower compared to the control group (Fig. 5c, d).

The obtained results indicate impaired and slowed development of sphenoid sinuses in CF patients.

Similar results were found for the frontal sinuses measurements. There is a statistically significant difference between the volumes of the frontal sinuses when comparing the CF group with the control group for all age groups over 6 years of age. The group of healthy children has a statistically larger frontal sinus volume compared to the study group (Fig. 4). The trend line in both groups is increasing with age, but this relationship is more pronounced for the healthy children group (Fig. 5e, f).

The development and the pneumatization process of the frontal sinuses do not begin until approximately 6–9 years of age. In younger children, only the frontal recesses are visible. For this reason, the results obtained for groups under 6 years of age are impossible to evaluate. The results of measurements for younger groups have been put on the charts. However, it should be remembered that the absence of frontal sinus under the age of 9 is the developmental norm [6, 8].

Based on the analyzed data, a statistically significant difference between the development of frontal sinuses of the study group and the control group can be observed. In the group of patients with CF, the volumes of the sinuses are lower. With age, their growth is observed, but at a lower rate than in healthy patients.

## Discussion

Cystic fibrosis negatively affects the development of the paranasal sinuses by slowing their natural volume expansion with age. The volume and the degree of sinus pneumatization in patients who require sinus surgery is a significant information for otorhinolaryngologists. Patients with CF due to impaired mucociliary drainage often require such treatment. The size of the sinuses increases significantly with age in the pediatric population. A surgeon performing sinus surgery in children is required to be aware of age-related changes in sinus volume.

The studies published to date on the development of maxillary sinuses in CF patients were conducted on small groups of patients [7]. The results revealed that the sinuses in children with CF were smaller than the control group, but statistical dependence was insignificant [9, 10]. Some reports of the studies on the subject of sinus volumes in CF patients do not include an age group discrimination, making it impossible to assess sinus development. Results of these studies also indicate smaller sinus volumes in patients with CF. Woodworth et al. evaluated the impact of the delta F508 gene mutation on sinus development in adult CF patients but without the measurements of their volumes [11]. A study on a larger group of CF patients was also performed, but the authors neither assessed the sinuses development nor the sinuses volume, but solely the severity of the lesions [12–14].

Some interesting studies concerning the development of the paranasal sinuses have been carried out on animals [15], on porcine models and in small study groups. Also, an evaluation of maxillary sinus development was carried out on small groups of animals (laboratory rats) comparing the volume between animals with and without mutation in the CFTR gene [16].

Studies on sinus development in healthy children were conducted by several authors, but the development in children with CF was not evaluated [8, 17–23]. Research conducted by some authors on sinus expansion has important implications for evaluating the process of their volume increase in healthy children [6]. The largest collected group of healthy pediatric patients for maxillary sinus volume measurements of 1,452 individuals was collected by Adibelli et al. [24]. Unlike other researchers, they made these measurements based on magnetic resonance imaging (MRI). Some studies of sinus volume measurements have been performed in South Africa [25] or Asia [26, 27]. However, they did not include patients with CF. Differences in sinus volume depending on race can occur in healthy individuals and CF patients as well. The highest incidence of CF occurs in Caucasians with a frequency of 1:3,200 individual [28]. In people of African descent, the frequency is 1:15,000, and 1:35,000 in Asians [29]. Because the disease is most prevalent in Caucasian patients, measurements in this population are more relevant.

Based on the results of our analysis, an obvious connection between CF disease and its negative effects on sinus development was confirmed. Other authors studying this topic had a similar observation, but as mentioned before, their studies covered smaller groups, due to which a higher error probability may be associated with the results [7, 9, 10]. Our study revealed statistically significant differences between the volumes of the paranasal sinuses in children with CF compared to the healthy control group. CF has a significant impact on the development of the paranasal sinuses in children. It interferes with the process of increasing pneumatization and volume of the paranasal sinuses with age. Impaired mucociliary drainage is the source of mucus retention in the sinuses. Retained secretions affect the process of volume expansion, while reduced sinus pneumatization can lead to impaired ventilation and chronic inflammation. Persistent mucus and difficulties with its drainage, as well as chronic inflammation, often cause pathological sinus distension. However, this does not increase their total volume, according to our results,

A limitation of our study is the inability to measure the ethmoid sinuses. This is mainly due to previous surgical procedures on the sinus in children with CF, so that most ethmoid cells were permanently removed and it was impossible to make accurate measurements. Another important limitation is also the small study group in the youngest age group. However, this is due to the lack of sinus symptoms in children under the age of 3, and therefore no need to examine them with CT scans.

The designed research was to evaluate healthy children and 2 groups of patients with impaired mucociliary drainage. A third group of patients suffering from primary ciliary dyskinesia was also analyzed during the study. However, due to the small study group (a total of 15 tomography findings) and the lack of analysis on this basis, and the similar results already published for such a group size [30], these results were omitted from the manuscript and are included in the supplementary material (Table S1).

In summary, there is a significant difference in paranasal sinuses volumes between the healthy children and children with CF. The obtained results provide valuable knowledge on the impact of the CF on sinuses development. CF strongly affects the development of the paranasal sinuses, significantly hampering the volume growth as compared to healthy population. The results of the study conducted by our team provide more accurate data on a large number of patients (a significant percentage of the total population). Because of this, they are more reliable and more accurately represent the condition of the sinuses of CF patients. This may be important in understanding the progression of the disease and its influence on the quality and length of life of patients Thanks to this analysis, we can understand more about cystic fibrosis, which can contribute to improving diagnosis and therapy for patients with chronic sinusitis associated with CF. The results obtained in the study can also be used to determine developmental norms for children with CF.

### Supplementary Information

Below is the link to the electronic supplementary material.

Supplementary file1 (DOCX 16 KB)

## Data Availability

The authors confirm that the data supporting the findings of this study are available within the article and its supplementary materials.
