# Supplementary material for: The development of paranasal sinuses in patients with cystic fibrosis: sinuses volume analysis
Source: Eur Arch Otorhinolaryngol. 2023 Oct 26;281(2):795–803. doi: 10.1007/s00405-023-08236-x (PMC10796702; doi:10.1007/s00405-023-08236-x)
Supplement: Supplementary file 1 — Supplementary file1 (DOCX 16 KB) [file 405_2023_8236_MOESM1_ESM.docx]

| N  [CT scans] | Age  [year] | Right maxillary sinus [cm^3^] | Left maxillary sinus  [cm^3^] | Sphenoid sinus  [cm^3^] | Right frontal sinus  [cm^3^] | Left frontal sinus  [cm^3^] |
| --- | --- | --- | --- | --- | --- | --- |
| 1 | 1.4 | 10.96 | 11.25 | 11.42 | 3.00 | 5.54 |
| 2 | 3.6 | 4.67 | 4.73 | 1.60 | 0.00 | 0.00 |
| 3 | 5.1 | 12.48 | 12.97 | 13.96 | 3.45 | 5.82 |
| 4 | 6.1 | 9.31 | 8.85 | 4.17 | 0.15 | 0.21 |
| 5 | 7.3 | 9.33 | 9.81 | 3.74 | 0.73 | 0.60 |
| 6 | 10.2 | 10.14 | 9.44 | 6.52 | 0.17 | 1.08 |
| 7 | 11.4 | 12.17 | 10.34 | 11.91 | 5.45 | 3.78 |
| 8 | 12.6 | 9.86 | 9.44 | 3.12 | 2.42 | 2.75 |
| 9 | 13.4 | 14.79 | 13.90 | 13.52 | 2.59 | 2.21 |
| 10 | 13.7 | 19.96 | 17.76 | 3.68 | 4.55 | 5.32 |
| 11 | 14.6 | 11.16 | 10.35 | 3.71 | 3.31 | 3.59 |
| 12 | 14.7 | 17.54 | 16.45 | 12.04 | 1.32 | 4.07 |
| 13 | 15.4 | 9.50 | 8.70 | 1.50 | 0.33 | 0.75 |
| 14 | 17.1 | 19.00 | 16.77 | 10.22 | 11.97 | 11.35 |
| 15 | 17.9 | 11.82 | 8.85 | 2.37 | 0.78 | 1.02 |

Table S1. Summary of PCD group sizes for each sinus N – number of scans
